# The role of preoperative lymphoscintigraphy in sentinel lymph node identification among patients with breast cancer: a retrospective study

**DOI:** 10.25122/jml-2025-0177

**Published:** 2026-05

**Authors:** Samia Abdelgauom Fathelrahman, Taifor Ahmed Albosairi, Amira Mabkhot Balobaid, Maram Awed Al-Korbi, Fayroz Mahmood Hafiz, Reema Ali Alkorbi, Rahaf Yasir Al-Saydah, Sawsan Ahmed Osman

**Affiliations:** 1 Radiology Department, College of Applied Medical Sciences, Najran University, Saudi Arabia; 2 Nuclear Medicine Sciences, Sudan University of Sciences and Technology, Khartoum, Sudan

**Keywords:** preoperative lymphoscintigraphy, sentinel lymph node (SLN), breast cancer, BC, breast cancer, SLNs, Sentinel Lymph Nodes, KKH, King Khalid Hospital, SLNB, Sentinel Lymph Node Biopsy, KSA, Kingdom of Saudi Arabia, SPSS, Statistical Package for Social Sciences

## Abstract

Breast cancer (BC) is the leading cause of death from cancer among women worldwide. The principle of lymphoscintigraphy is to image radiotracers injected into the interstitial space and follow their transport through the initial lymphatics, the lymphatic vessels, and lymph nodes to the main lymphatic ducts, and finally to the liver. The objective of the study was to identify the role of preoperative lymphoscintigraphy in sentinel lymph node (SLN) identification among patients with breast cancer. A retrospective single-center study was conducted at King Khalid Hospital using the PACS system. The study included 72 adult female patients diagnosed with breast cancer between February and May 2025 who underwent lymphoscintigraphy before SLN biopsy. The results showed that 36.7% of patients were aged 51–70 years, and 56.7% had tumors in the right breast. Most patients (86.7%) had negative SLN results, while 13.3% were positive for metastasis. More than half of the patients (66.7%) had small tumors measuring 1–2 cm. Regarding surgical management, 66.7% underwent breast lumpectomy, 13.3% underwent mastectomy, and 20% had not yet undergone surgery. A weak correlation was observed between SLN results and the type of operation (*P* = 0.046), although the strength of association was low. There was also a weak and non-significant correlation between SLN status and tumor size (*P* = 0.227). We conclude that lymphoscintigraphy plays an important role in identifying sentinel lymph nodes and helps minimize postoperative complications.

## Introduction

Approximately 75–80% of lymphatic drainage from the breast flows to the axillary lymph node group, which serves as the primary drainage pathway. Most of this lymph is directed to the pectoral (anterior) axillary nodes. The remaining 20–25% drains into the internal mammary lymph nodes [1]. Breast neoplasms can be benign (fibroadenoma) or malignant (breast cancer), depending on the type. A breast lump is the typical presentation for either scenario. Approximately 7% of breast lumps are fibroadenomas, around 10% are malignant (breast cancer), and the remaining cases are attributed to other benign conditions or no significant pathology [2]. Breast cancer (BC) is the second leading cause of cancer-related death among women in the United States and the leading cause of cancer-related mortality among women worldwide [3]. BC accounts for 30% of all female cancer diagnoses, with 276,480 expected new cases and over 42,000 predicted deaths in 2020, according to Cancer Statistics 2020 [4].

The lymphatic system plays a crucial role in returning excess interstitial fluid and proteins from tissues back to the circulatory system. It is also an essential component of the immune system, transporting lymph and foreign substances such as bacteria and cancer cells to lymph nodes, where immune responses are initiated. When lymph vessels, ducts, nodes, or lymphatic tissues become blocked, infected, inflamed, or invaded by cancer cells, the function of the lymphatic system can be impaired. Scintigraphic evaluation of the lymphatic system is commonly used in patients with suspected lymphedema and in cancer patients to identify sentinel lymph nodes [5].

One way to map the lymphatic system is by lymphoscintigraphy. After the thoracic duct and cisterna chyli were discovered in the 1600s, efforts to understand and map the lymphatic system began [6]. The principle behind lymphoscintigraphy is to image radiotracers injected into the interstitial space and track their movement through lymph nodes, lymphatic capillaries, and early lymphatics before reaching the main lymphatic ducts and the liver. Focused tracer buildup outside of vessels and cutaneous backflow are the most significant results to be aware of, as they are pathognomonic for lymphedema. Depending on the tumor type, there are many ideal injection techniques for radiotracer use in sentinel lymph node (SLN) imaging. As a general rule, a sentinel lymph node is the first visible lymph node that is closest to the injection site and has the highest radiotracer uptake. The sentinel lymph node is the first lymph node to evacuate lymph from the primary tumor, as well as any metastatic cancer cells [7].

### Lymphoscintigraphy

The sentinel lymph node is defined as the first lymph node, or group of nodes, that drains lymph from a primary malignancy. It is theorized that in instances of confirmed malignant spread, cancer cells that metastasize from the tumor primarily focus on the sentinel lymph nodes as their preferred site. The process of identifying, excising, and analyzing the sentinel lymph nodes associated with a particular tumor is known as the sentinel node procedure (also referred to as sentinel lymph node biopsy (SLNB) [8]. Typically, this procedure is finalized several hours before the biopsy is conducted. Similarly, the physician administers an injection of blue dye approximately 15 minutes before the biopsy commences. Subsequently, during the biopsy, the physician utilizes a gamma probe or a Geiger counter to identify which lymph nodes have absorbed the radio nuclide, in addition to visually inspecting the lymph nodes for any discoloration. One or more nodes may absorb the dye and radioactive tracer, which are then classified as the sentinel lymph nodes. The surgeon subsequently excises these lymph nodes and submits them to a pathologist for expedited analysis under a microscope to ascertain the presence of malignancy. After radiotracer injection, scintigraphic imaging generally begins within 5 minutes, and the sentinel node is usually visualized within 5–60 minutes. Generally, this process is completed several hours before the biopsy is performed [9].

Recent advancements in ultrasound technology have improved diagnostic confidence in identifying benign breast conditions, thereby reducing unnecessary biopsies. Magnetic resonance imaging (MRI) is also valuable for screening high-risk patients, further evaluating indeterminate findings, and conducting preoperative assessment in advanced breast cancer. MRI can detect additional lesions that may alter the surgical plan, such as converting a breast-conserving lumpectomy to a mastectomy when appropriate [10].

## Material and methods

This retrospective hospital-based study was conducted at King Khalid Hospital (KKH) in Najran Province, Kingdom of Saudi Arabia. Patient data were retrieved from medical records and the Picture Archiving and Communication System (PACS). The study included adult female patients diagnosed with breast cancer who underwent preoperative lymphoscintigraphy before sentinel lymph node biopsy. The study was carried out between February and May 2025 and included 72 female patients aged between 20 and 70 years. Pregnant women were excluded from this study.

Demographic and clinical data collected included age, side of breast lesion, tumor size, SLN results, and type of surgical procedure performed. All data were obtained from patient files and hospital records at KKH. Statistical analysis was performed using SPSS version 26 to evaluate the influence of lymphoscintigraphy findings on patient management.

All required imaging studies and diagnostic tests were reviewed to confirm the diagnosis and determine the cancer stage. For lymphoscintigraphy, the radiotracer was injected either superficially or deeply, depending on the selected technique. Superficial injection methods included intradermal, subcutaneous, subareolar, and periareolar approaches, while deep techniques included peritumoral or intratumoral injections. Subcutaneous, periareolar, and subareolar injections are generally associated with a lower detection rate of non-axillary nodes.

The combination of radiocolloid injection and blue-dyed injection immediately prior to surgery provides the highest sensitivity and specificity of the technique. Nevertheless, numerous studies have also demonstrated that SLN biopsy using radiotracer alone is effective. The material was initially injected near the tumor. The injected material was located either visually or with a device that detects radioactivity. The sentinel node(s), which are the first lymph node(s) to absorb the material, were excised and examined for cancer cells. Images were typically obtained using a planar gamma camera equipped with a large-field-of-view detector, a parallel-hole collimator, and an energy setting suitable for Tc-99m (140 keV). Dynamic and/or static acquisitions were conducted for 45 minutes to an hour post-injection, utilizing a cobalt-57 flood source as necessary to outline the body.

## Results

Early detection of breast cancer greatly increases cure and survival rates, as well as expands treatment options and improves their effectiveness. This study aimed to evaluate the impact of lymphoscintigraphy in lymphatic mapping and SLN identification in breast cancer patients, which can significantly influence the type of surgery a patient undergoes. Statistical data were obtained from the Radiology Department.

Table 1 shows that more than one-third of patients (*n* = 26) were aged 51–70 years, and one-third (*n* = 24) were aged 41–50 years. Additionally, 12 patients were aged 20–30 years, while only ten patients were aged 31–40 years.

**Table 1:** Distribution of patients according to age

Characteristics	Percentage	Frequency	Age group
**Age Groups**	16.7%	12	20–30
	13.3%	10	31–40
	33.3%	24	41–50
	36.7%	26	51–70
	100%	72	Total

Table 2 shows the distribution of patients according to the side of the breast affected by the tumor. More than half of the participants (*n* = 41; 57%) had tumors in the right breast, while the remaining patients (*n* = 31; 43%) had tumors in the left breast.

**Table 2:** Distribution of breast side tumor

Side of the breast	Number	Percentage
Right	41	57%
Left	31	43 %
Total	72	100%

Table 3 shows the distribution of SLN identification and localization groups during the examination. Half of the patients (*n* = 36; 50%) had SLN identification in one group, while 33.3% (*n* = 24) had SLN identified in two groups. The remaining 16.7% (*n* = 12) had SLN identified in three groups.

**Table 3:** Distribution of SLN identification and localization groups

Percentage	Number	SLN
1 group	36	50%
2 group	24	33.3 %
3 group	12	16.7%
Total	72	100%

Table 4 shows the distribution of patients by SNLB findings. Most patients (*n* = 62; 86.2%) had negative SLN results, while only 13.8% (*n* = 10) had positive SLN results.

**Table 4:** Distribution of patients according to the SLN biopsy finding

Type of result	Number	Percentage
Positive	10	13.8%
Negative	62	86.2%
Total	72	100%

Table 5 shows the distribution of patients according to the type of operation. More than half of the patients (*n* = 48; 66.7%) underwent breast lumpectomy, including 18 patients (37.5%) who had SLN dissection. In addition, 13.3% of the patients (*n* = 10) underwent breast mastectomy, and 20.0% (*n* = 14) had not yet undergone surgery.

**Table 5:** Distribution of patients according to the type of operation

Type of operation	Number	Percentage
Breast mastectomy	10	13.3%
Breast lumpectomy	48 (18 of them; 37.5% with SLN dissection)	66.7%
Not yet performed	14	20.0%
Total	72	100%

Table 6 shows the distribution of patients according to tumor size. Most patients (*n* = 48; 66.7%) had tumors measuring 1–2 cm, while the remaining patients (*n* = 24; 33.3%) had tumors larger than 2 cm.

**Table 6:** Distribution of patients according to the tumor size

Size of tumor	Number	Percentage
1–2 cm	48	66.7%
More than 2 cm	24	33. 3%
Total	72	100%

Table 7 shows the correlation between SNL results and the type of operation; however, the correlation is small (*P* value = .046). Also, there was a small correlation between SLN and tumor size (*P* value = .227).

**Table 7:** Correlation between SNL, the type of operations & the size of tumor

Correlations				
		Type of operation	Size of tumor	SNL results
Type of operation	Pearson Correlation	1	-.082-	.046
	Sig. (2-tailed)		.666	.811
	n	72	72	72
Size of tumor	Pearson Correlation	-.082-	1	.277
	Sig. (2-tailed)	.666		.138
	n	72	72	72
SNL results	Pearson Correlation	.046	.277	1
	Sig. (2-tailed)	.811	.138	
	n	72	72	72

*P*-values are considered statistically significant at < 0.05

## Discussion

Breast cancer is the most common cancer and the second leading cause of cancer-related death among women worldwide. According to Cancer Statistics 2020, breast cancer accounts for 30% of female cancers, with an estimated 276,480 new cases and more than 42,000 deaths reported in 2020. In this study, we discussed the importance and impact of lymphoscintigraphy in lymphatic mapping and SLN identification in breast cancer patients, which can significantly affect the type of surgery that a woman undergoes.

Regarding age distribution, Table 1 indicates that 36.7% of participants were between 51 and 70 years of age. Similar results were reported by Jamaris *et al*. [11], who found that more than half of the patients (54.6%) were aged 55 years or older.

Regarding the affected side of the breast, Table 2 shows that more than half of the patients (56.7%) had tumors in the right breast, while 43.3% had tumors in the left breast. These findings are consistent with a study conducted by Bakhtiar *et al*. [12], which reported that 62% of patients had carcinoma in the right breast, whereas 38% had carcinoma in the left breast.

According to the number of SLNs identified by lymphoscintigraphy, Table 3 shows that one SLN group was identified in 50% of patients, two SLN groups in 33.3%, and three SLN groups in 16.7%. These results reflect that lymphoscintigraphy is effective and highly reliable in localizing sentinel lymph nodes in breast cancer patients.

Anwar *et al*. [13] reported that the majority of patients had three SLN groups, accounting for 54.2%. Additionally, 26.3% of patients had two SLN groups, while only 0.9% had one SLN group visualized on lymphoscintigraphy.

Regarding the SLN results, Table 4 indicates that most patients (86.7%) had negative SLN metastasis, while only 13.3% had positive SLN metastasis. Our results are consistent with those of Veronesi *et al*. [14], who reported that 32% of patients had positive SLN results and 68% had negative SLN results. However, another study by Bakhtiar *et al*. [12] reported different findings, showing that SLN was positive for carcinoma in 61 of 85 patients (71.7%), while only two patients had negative sentinel lymph nodes.

Regarding the type of operation, Table 5 shows that more than half of the patients (66.7%) underwent breast lumpectomy. Among them, 37.5% underwent SLN dissection, while the remaining patients did not. In addition, 20% of the patients had not yet undergone surgery, while 13.3% underwent total breast mastectomy. These findings suggest that most patients were diagnosed at an early stage of the disease. However, our results differ from those reported by Koka *et al*. [15], who found that mastectomy (58.2%) was the most common type of surgery.

Regarding tumor size, Table 6 shows that more than half of the patients (66.7%) had tumors measuring 1–2 cm, while one third of the patients (33.3%) had tumors larger than 2 cm. Our findings differ from those reported by La Verde *et al*. [16], who found that most patients (75.7%) had small tumors (<20 mm), with a mean tumor size of 15.7 mm (range 1.0–50.0 mm).

Regarding Table 7, the study found a correlation between SNL and the type of operation, but it was not significant (*P* = .046). The results also showed a correlation between SNL and tumor size, but it was not significant (*P* = .227). Our results agree with a study by La Verde *et al*. [16], which showed that a positive SLN was significantly associated with tumor size (p = 0.004). Another study by Fujii *et al*. [17] agrees with our study and suggests that the presence of lymphatic invasion, combined with cancer size, could be considered a strong risk factor for SLN. However, their result was significantly associated.

## Conclusion

At the end of the study, the research concludes the following results:

1. Lymphoscintigraphy is an effective method for identifying and localizing sentinel lymph nodes and assessing tumor metastasis before breast surgery.
2. Most patients had a breast lumpectomy without SLN dissection.
3. Most of the patients had negative SLN results.
4. Most patients had small tumor sizes.
5. A correlation was observed among SLN status, operation type, and tumor size; however, it was not statistically significant.
